# Supplementary figures and images for: Reliability of gamified reinforcement learning in densely sampled longitudinal assessments
Source: PLOS Digit Health. 2023 Sep 6;2(9):e0000330. doi: 10.1371/journal.pdig.0000330 (PMC10482292; doi:10.1371/journal.pdig.0000330)

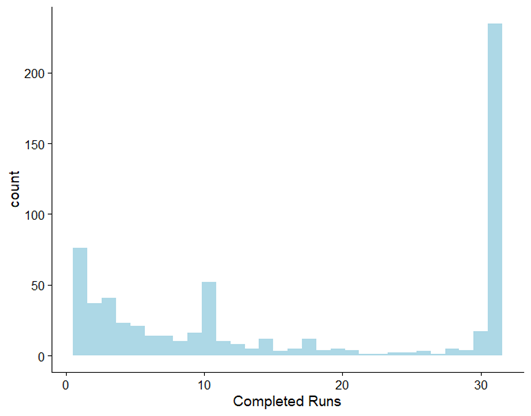

Supplement: S1 Fig — (TIF) [file pdig.0000330.s001.tif]

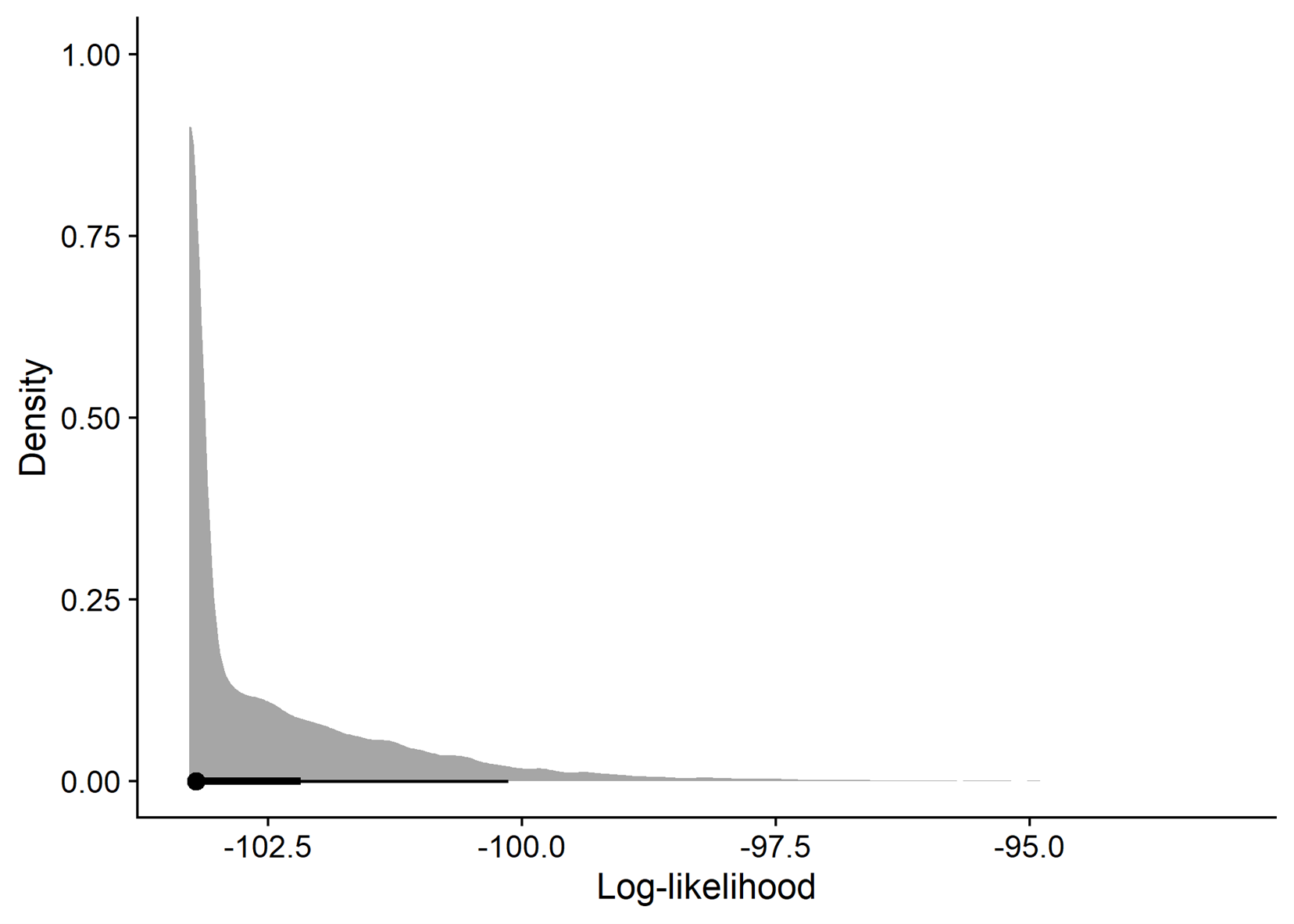

Supplement: S2 Fig — We only included runs with a low chance of coming from random choices (i.e., Log-likelihoods higher than the 95 percentile from this random distribution, -100.13). (TIF) [file pdig.0000330.s002.tif]

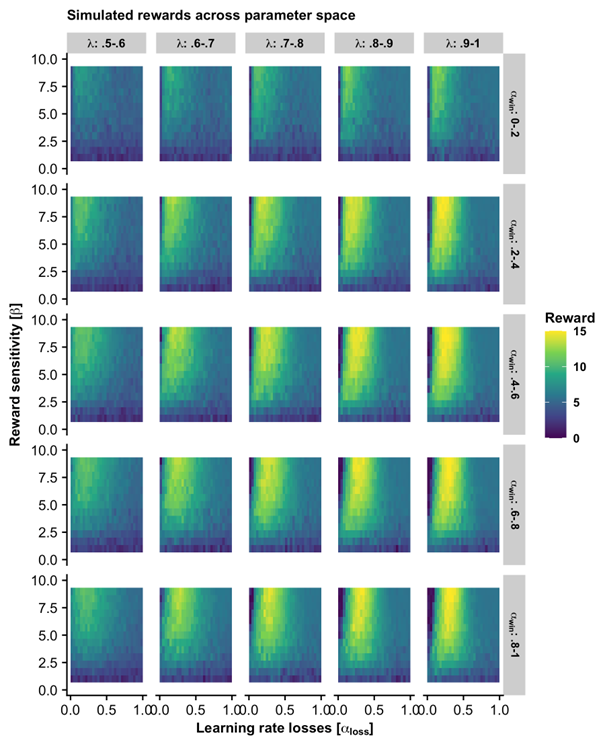

Supplement: S3 Fig — The learning rate for wins has less influence on the obtained rewards apart from very low learning rates. (TIF) [file pdig.0000330.s003.tif]

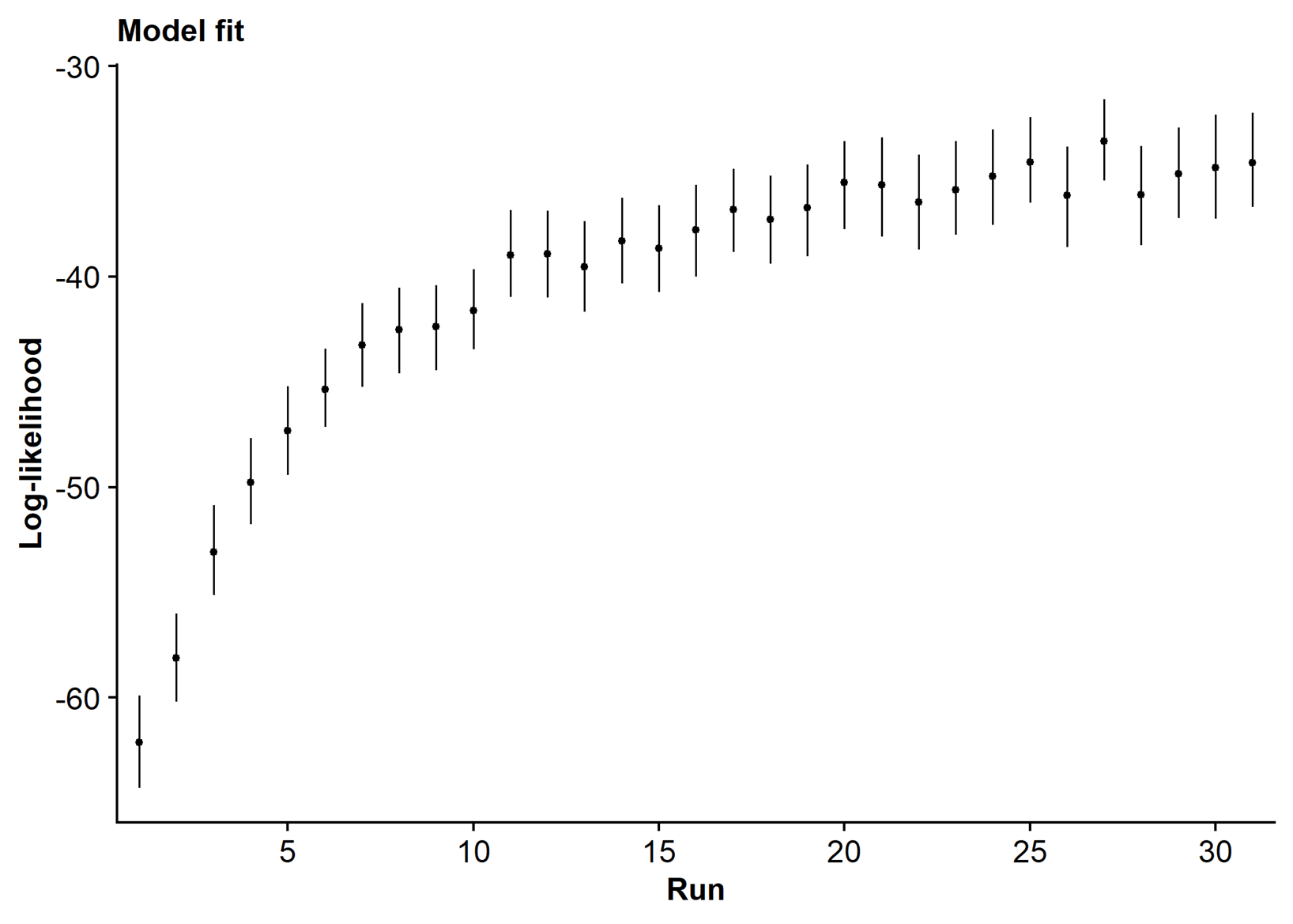

Supplement: S4 Fig — (TIF) [file pdig.0000330.s004.tif]

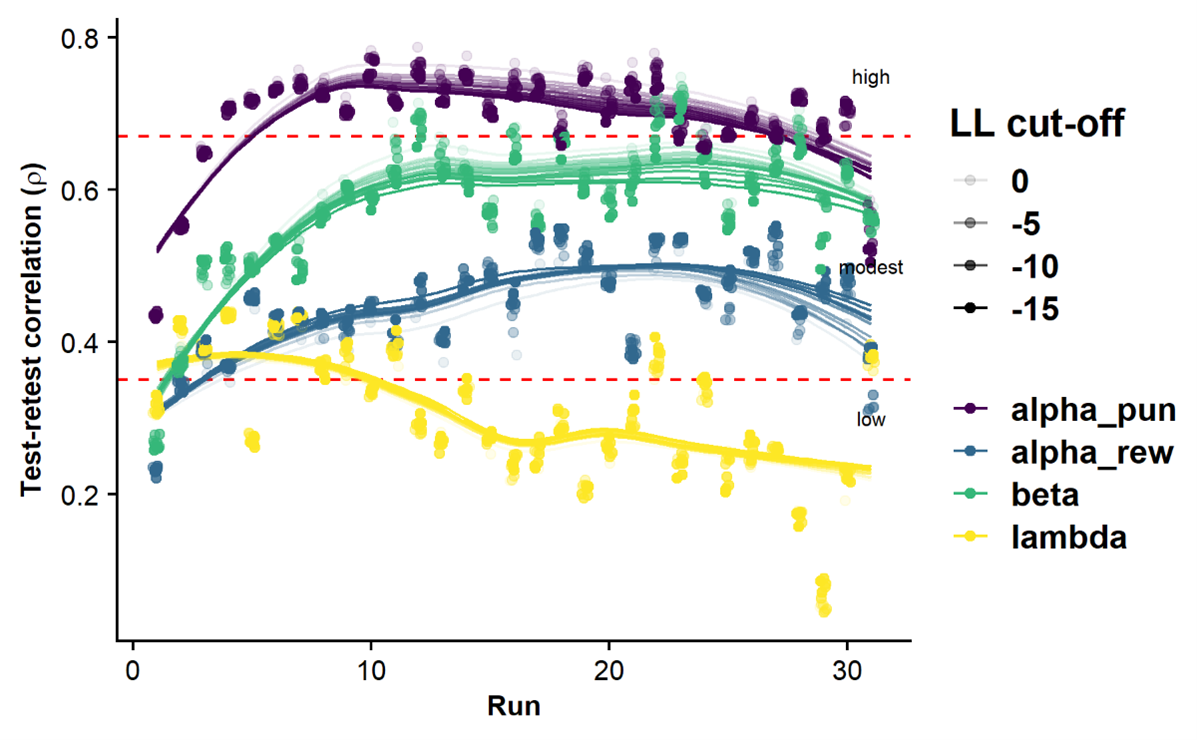

Supplement: S5 Fig — The patterns across runs and between parameters remain comparable across all exclusion criteria. (TIF) [file pdig.0000330.s005.tif]

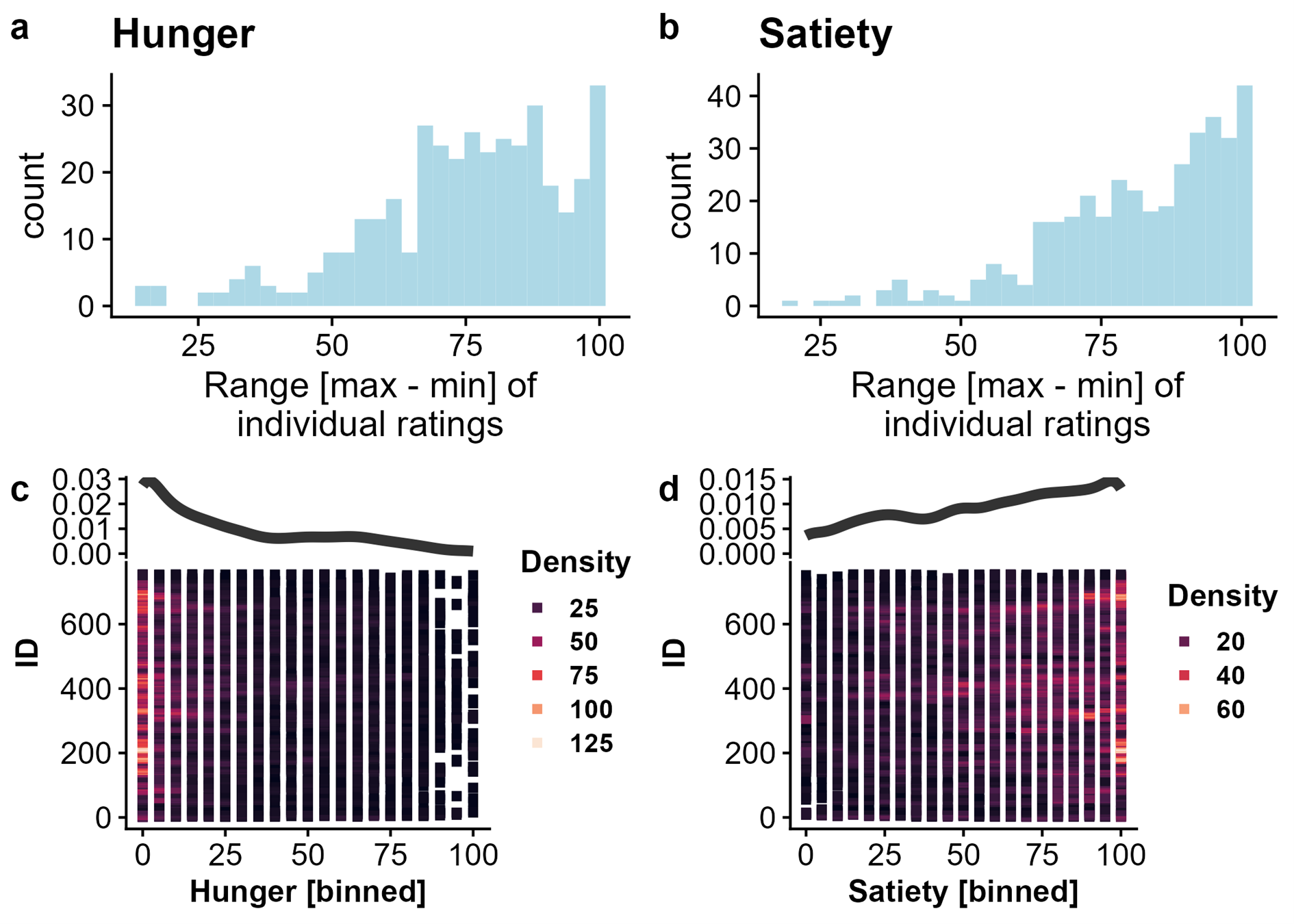

Supplement: S6 Fig — a)-b) distribution of individual ranges (i.e., maximal value–minimal value) of hunger (a) and satiety (b) ratings show that more than 90% participants had ranges exceeding 50 or 60 points for hunger and satiety, respectively. c)-d) Density of run-based hunger and satiety ratings for each participant show that large areas of possible metabolic states are covered in most participants. (TIF) [file pdig.0000330.s006.tif]
