# Supplementary material for: Reliability of gamified reinforcement learning in densely sampled longitudinal assessments
Source: PLOS Digit Health. 2023 Sep 6;2(9):e0000330. doi: 10.1371/journal.pdig.0000330 (PMC10482292; doi:10.1371/journal.pdig.0000330)
Supplement: S1 Table — (DOCX) [file pdig.0000330.s007.docx]

**S1 Table:** Demographic and psychometric information on the sample

| Variable | N | >= 10 valid runs,  N = 384 | < 10 valid runs,  N = 8 |
| --- | --- | --- | --- |
| **Sex** | 3 |  |  |
| female |  | 291 (82%) | 3 (75%) |
| male |  | 61 (17%) | 1 (25%) |
| other |  | 1 (0.3%) | 0 (0%) |
| **Age** | 3 |  |  |
| Mean (SD) |  | 34.7 (14.1) | 44.0 (16.6) |
| Median (IQR) |  | 29.0 (23.0, 48.0) | 45.0 (34.5, 54.5) |
| Range |  | 18.0, 68.0 | 24.0, 62.0 |
| **Education** | 3 |  |  |
| student |  | 4 (1.1%) | 0 (0%) |
| no college degree |  | 5 (1.4%) | 0 (0%) |
| at least 8 years |  | 51 (14%) | 1 (25%) |
| at least 12 years |  | 162 (46%) | 0 (0%) |
| college degree |  | 123 (35%) | 2 (50%) |
| other |  | 8 (2.3%) | 1 (25%) |
| **BMI** | 3 |  |  |
| Mean (SD) |  | 26.4 (7.1) | 27.4 (4.9) |
| Median (IQR) |  | 24.2 (21.3, 29.8) | 27.1 (24.2, 30.3) |
| Range |  | 13.8, 58.5 | 22.2, 33.3 |
| **BDI** | 3 |  |  |
| Mean (SD) |  | 11.0 (10.3) | 16.8 (8.6) |
| Median (IQR) |  | 8.0 (3.0, 15.0) | 17.0 (13.5, 20.2) |
| Range |  | 0.0, 49.0 | 6.0, 27.0 |
| **BDI categories** | 3 |  |  |
| no depression |  | 249 (71%) | 1 (25%) |
| mild depression |  | 42 (12%) | 2 (50%) |
| moderate  depression |  | 33 (9.3%) | 1 (25%) |
| **AUDIT** |  |  |  |
| Mean (SD) |  | 3.5 (3.3) | 3.2 (2.2) |
| Median (IQR) |  | 3.0 (1.0, 5.0) | 3.0 (1.8, 4.5) |
| Range |  | 0.0, 17.0 | 1.0, 6.0 |
| **CAARS_DSM_sum** |  |  |  |
| Mean (SD) |  | 4.0 (3.1) | 7.2 (1.7) |
| Median (IQR) |  | 4.0 (1.0, 6.0) | 7.5 (6.5, 8.2) |
| Range |  | 0.0, 12.0 | 5.0, 9.0 |
| **FTND** | 3 |  |  |
| 0 |  | 334 (95%) | 4 (100%) |
| 1 |  | 12 (3.4%) | 0 (0%) |
| 3 |  | 7 (2.0%) | 0 (0%) |
| **LSAS_score** | 3 |  |  |
| Mean (SD) |  | 37.7 (24.5) | 44.0 (15.6) |
| Median (IQR) |  | 33.0 (20.0, 52.0) | 42.0 (31.5, 54.5) |
| Range |  | 0.0, 123.0 | 30.0, 62.0 |
|  |  |  |  |
| **Trait Anxiety** |  |  |  |
| Mean (SD) |  | 42.8 (12.2) | 53.0 (17.1) |
| Median (IQR) |  | 41.0 (33.0, 52.0) | 50.5 (44.8, 58.8) |
| Range |  | 21.0, 74.0 | 35.0, 76.0 |
| **YFAS_sum** |  |  |  |
| Mean (SD) |  | 19.8 (14.5) | 48.5 (24.1) |
| Median (IQR) |  | 16.0 (8.0, 29.0) | 52.0 (35.2, 65.2) |
| Range |  | 0.0, 68.0 | 18.0, 72.0 |
| **YFAS_diagnosis** |  | 70 (20%) | 2 (50%) |
| **missing** | 3 |  |  |
| available |  | 353 (92%) | 4 (50%) |
| missing |  | 31 (8.1%) | 4 (50%) |

Note: BMI = Body mass index, BDI = Becks Depression Inventory, AUDIT = Alcohol Use Disorder Identification Test, Conners’ Adult ADHD Rating Scales, FTND = Fagerström Test for Nicotine Dependence, Liebowitz Social Anxiety Scale, YFAS = Yale food addiction scale, SD = Standard deviation, IQR = Interquartile range
