# Supplementary material for: Reliability of gamified reinforcement learning in densely sampled longitudinal assessments
Source: PLOS Digit Health. 2023 Sep 6;2(9):e0000330. doi: 10.1371/journal.pdig.0000330 (PMC10482292; doi:10.1371/journal.pdig.0000330)
Supplement: S2 Table — (DOCX) [file pdig.0000330.s008.docx]

## **Table S2** Reliability measures for participants with BDI >= 14 vs. BDI < 14 and participants with vs. binge eating disorder

| Measures | ICC_unc_ | ICC_cond_ | ICC_unc_ | ICC_cond_ | ICC_unc_ | ICC_cond_ | ICC_unc_ | ICC_cond_ |
| --- | --- | --- | --- | --- | --- | --- | --- | --- |
|  | BDI < 14  N = 248 | | BDI >= 14  N = 104 | | No BED N = 268 | | BED N = 40 | |
| **Model parameters** |  |  |  |  |  |  |  |  |
| Log-likelihood | .43 | .47 | .42 | .45 | .43 | .47 | .35 | .39 |
| Learning rate loss | .53 | .55 | .54 | .56 | .54 | .56 | .48 | .53 |
| Learning rate win | .22 | .22 | .26 | .26 | .24 | .25 | .23 | .24 |
| Reward sensitivity | .34 | .36 | .32 | .33 | .33 | .34 | .31 | .32 |
| Lambda | .37 | .38 | .43 | .43 | .28 | .29 | .55 | .57 |

*Note*: SD = standard deviation, ICC = intraclass correlation coefficient
