# Supplementary material for: Reliability of gamified reinforcement learning in densely sampled longitudinal assessments
Source: PLOS Digit Health. 2023 Sep 6;2(9):e0000330. doi: 10.1371/journal.pdig.0000330 (PMC10482292; doi:10.1371/journal.pdig.0000330)
Supplement: S1 Appendix — (PDF) [file pdig.0000330.s009.pdf]

S1 Appendix

Models with multiplicative weighting

Instead of using an additive weighting as in our final model, the integration of reward magnitude and win probability has often been implemented as a multiplicative weighting (Behrens et al., 2007). Here, an individual chooses the option that maximizes the expected outcome by scaling the win probability of each option with its associated reward into action weights (*W*). These action weights are weighted by a parameter, γ, that indicates whether choices either over- (γ < 1) or undervalue (y > 1) options with high reward magnitudes compared to an integration of both reward magnitude and win probability with equal importance (Behrens et al., 2007).

$$W_t\ (Option\ A) = \mathrm{F}(p_{win,t}\ (Option\ A),\ \gamma) * f(Option\ A) \quad \text{and} \quad (4)$$

$$W_t\ (Option\ B) = \mathrm{F}((1 -\ p_{win,t}\ (Option\ A)),\ \gamma) * f(Option\ B) \quad (5)$$

Here, F is a linear transform that scales the win probability according to the individual level of valuing the reward magnitude yet ensures that it remains within the bounds of 0 and 1.

$$F\left(p_{win,t}, \gamma\right) = \max\left[\min\left[\left(\gamma * \left(p_{win,t}(Option\ A) - 0.5\right) + 0.5\right), 1\right], 0\right] \quad (6)$$

In that case, decision-making with equal weighting would be achieved with γ = 1, whereas γ > 1 would lead to decisions that are predominantly based on the learned probabilities and less on differences in reward points (i.e., avoiding the risk of choosing the wrong option). In contrast, γ < 1 would lead to decisions that are predominantly based on differences in reward points at the risk of choosing the wrong option.

Model Comparisons

We compared four different models (one learning rate and multiplicative weighting (1LR_gamma), two learning rates and, e.g., γ = 1 equal multiplicative weighting (2LR), two learning rates and free multiplicative weighting (2LR_gamma), and two learning rates and additive weighting (2LR_lambda)) by calculating the Bayesian information criterion (BIC) across all runs included in the analyses and additionally evaluated which model explained the data best in most runs, again using the BIC for each individual run. The winning model (BIC = 1,004,374, ΔBIC compared to less complex models between 92,585 – 242,904) included two learning rates and the additive weighting (Figure S3). Thus, we performed all following analyses using the model including two learning rates and the additive mixture parameter λ.

Test-retest reliability

The ICC describes the reliability of a measure on the scale of a correlation coefficient, where values close to 1 reflect high similarity within participants, whereas lower ICCs indicate lower similarity within participants. As described in Raudenbush and Bryk (2002), we derived the unconditional ICC based on the null model: Parameter ~ (1|ID) implemented with lme4 in R The ICC describes the reliability of a measure on the scale of a correlation coefficient, where values close to 1 reflect high similarity within participants, whereas lower ICCs indicate lower similarity within participants. with the formula:

$$ICC = \frac{\sigma_{u0}}{(\sigma_{u0} + \sigma_e)} \quad (8)$$

where σ_{uo} is the variance explained by the random intercept (ID) and σ_e denotes the residual variance. This ICC assesses absolute agreement and

corresponds to an ICC derived from a random effects model with repeated measures (ICC(1,k), (Koo & Li, 2016; Shrout & Fleiss, 1979)). Additionally, we calculated the conditional ICC taking (fixed) run effects (log-transformed) into account with the following mixed-effects model:

$$\text{Parameter} \sim 1 + \text{Run} + (1|\text{ID}). \quad (9)$$

This ICC assesses consistency when considering systematic differences between timepoints and corresponds to the ICC derived from a two-way mixed effects model with repeated measures (ICC(3,k), (Koo & Li, 2016; Shrout & Fleiss, 1979)). We interpreted the ICC according to recommendations by Shrout and Fleiss (1979) so that values < 0.4 reflect poor, values between 0.4 and 0.6 reflect fair, between 0.6 to 0.75 reflect good, and values > 0.75 reflect excellent reliability.

Changes across runs in participants starting before March 2020

Notably, behavior across runs (i.e., increases in performance indicated by higher average rewards ($t=5.9$, $p < .001$), model fit ($t=10.33$, $p < .001$), reward sensitivity β ($t=9.2$, $p <.001$), and reliance on learned reward probabilities λ ($t=3.03$, $p = .004$), as well as lower learning rates for losses αpun ($t=-4.0$, $p = .0003$).

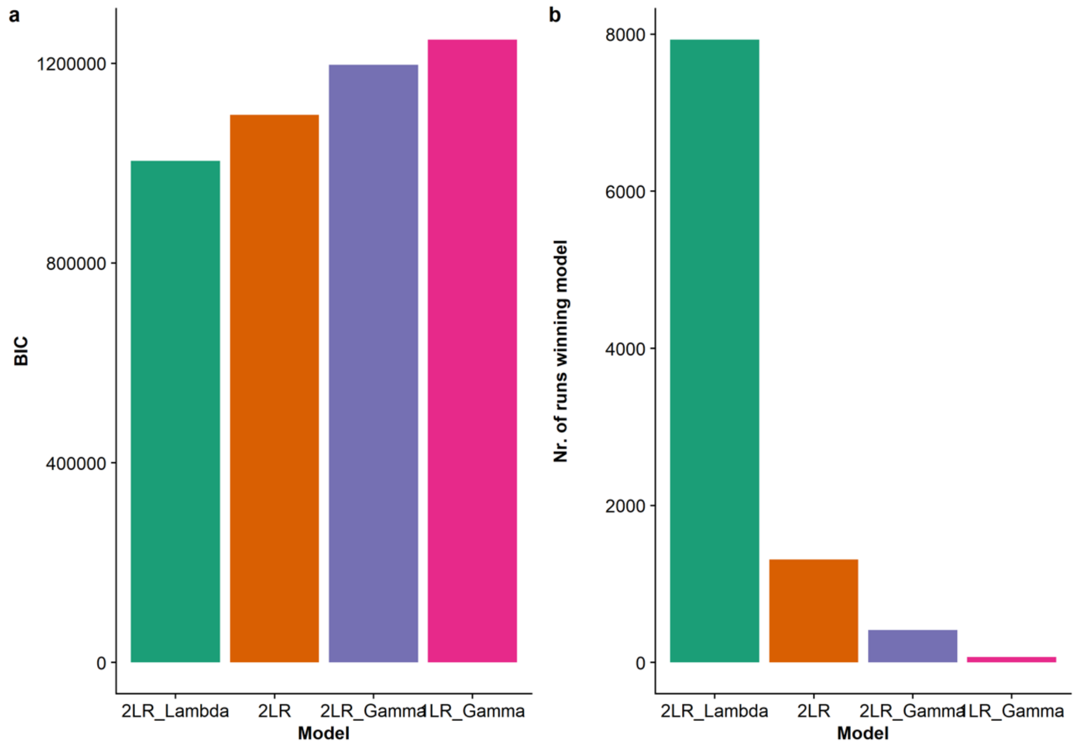

Figure A. Model comparisons of 4 candidate computational models revealed a model with 4 free parameters (learning rates for wins and losses, reward sensitivity, and an additive mixture parameter) to show the best model fit and lowest BIC across all runs (a) as well as in the highest number of single runs (b)

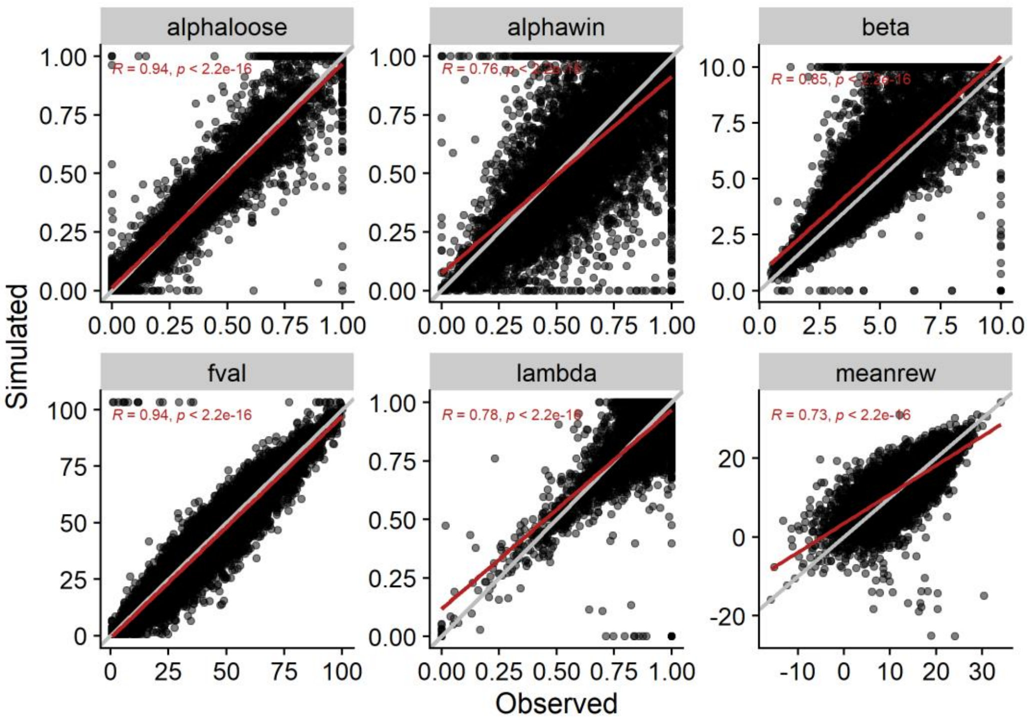

Figure B. Parameters in this 4 parameter model were successfully recovered from simulated data.

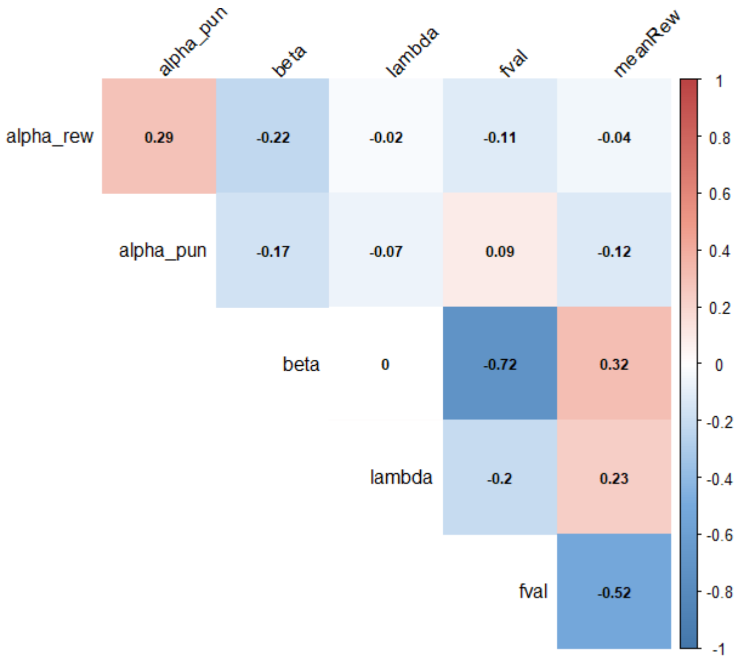

Figure C. Average reward is correlated with the model-fit (fval) as well as higher reward sensitivities (beta) and the weighting of learned win probabilities (Lambda). In contrast, learning rates especially for punishments are negatively associated with the average reward.

Table A. Reliability measures of the basic reinforcement learning model (Behrens et al., 2007)

Measures	Mean	SD	Median	10th Percentile	90th Percentile	ICC_{unc}	ICC_{cond}
**Model parameters**							
Log-likelihood	-56.6	21.7	-56.0	-84.2	-30.4	.32	.33
Learning rate	0.34	0.28	0.25	0.04	0.82	.53	.55
Reward sensitivity	2.74	1.47	2.58	1.39	4.10	.29	.29
Gamma	5.07	6.37	2.44	1.01	13.77	.27	.27

Note: SD = standard deviation, ICC = intraclass correlation coefficient
