# Supplementary material for: Reliability of gamified reinforcement learning in densely sampled longitudinal assessments
Source: PLOS Digit Health. 2023 Sep 6;2(9):e0000330. doi: 10.1371/journal.pdig.0000330 (PMC10482292; doi:10.1371/journal.pdig.0000330)
Supplement: Attachment — Submitted filename: ResponseLetter_v2.docx [file pdig.0000330.s010.docx]

Editor

I have only a few comments, and most of the points have been addressed by the reviewers.

1. As both reviewers have pointed out, the mathematical aspects of the publication should be made more accessible to a broader audience. It took me several attempts to read the publication and understand the mathematical background of different reinforcement learning models and their importance for this study

**We thank the editor for the encouraging and highly constructive feedback concerning our submission. We agree that the methods section contained a lot of specific information that was not tailored to a broader readership. Therefore, we now explain the final model in detail in the main body of the manuscript including the meaning of each parameter linking it to corresponding changes in behavior. To ensure replicability and transparency, we include all additional details for a narrower audience in the supporting information.**

2. I am curious to know which patient population would benefit from the game?

**We believe that gamified assessments of value-based decision-making and learning could be of broad use for a variety of patient populations. For example, monitoring the effects of a pharmacological treatment or cognitive behavioral therapy is important for clinical trials in patients with mental disorders, but they could also provide important insights into developing symptoms in neurological disorders, in addition to cognitive impairments that are tracked on a more routine basis already. Crucially, we show that psychometric properties improve with repeated assessments, indicating that such tools hold promise to provide relevant information about behavioral processes that have so far evaded longitudinal assessment at larger scales since they were limited to lab-based settings. Moreover, we have changed the title of the manuscript to: Reliability of gamified reinforcement learning in densely sampled longitudinal assessments to better represent its scope.**

**p.6:**

**To summarize, cross-sectional measures provide a snapshot of value-based decision-making, which cannot separate trait-like differences from state-like differences in behavior, specifically if reliability is low. To provide a much more comprehensive estimation of reliability in a clinically relevant setting, we present densely sampled longitudinal data of reinforcement learning and decision-making in a sample with diverse symptoms of psychopathology (open-source app Influenca: www.neuromadlab.com/en/influenca-2). To facilitate the investigation of altered value-based decision making in participants with pathological eating behavior (e.g., restrictive eating or binge eating) or mood and anxiety disorders, we developed a smartphone-based, gamified assessment. We measured decision-making over extended time periods in a large sample recruited to span a large range of body mass index (BMI) and with diverse symptoms of psychopathology. The longitudinal assessment across 30 runs enables us to investigate how reinforcement learning changes over runs and how variable behavior is within participants. Such a dense sampling approach provides important new information beyond a lab-based snapshot of behavior to characterize individual trajectories of behavior. For example, binge eating might be characterized by increased fluctuations in reward sensitivity are associated with higher fluctuations in reward learning (Neuser, Kühnel, Svaldi, & Kroemer, 2020). Here, we first evaluate the psychometric properties, such as reliability of behavioral parameters to ensure that the data provided by the app is able to provide reliable trait-like inter-individual differences while still capturing meaningful intra-individual variability. Such intra-individual variability may reflect potential adaptations in individual behavior, for example, in response to treatments, reflecting the range of the individual repertoire of behavior. Hence, more detailed knowledge of the psychometric characteristics of these parameters will provide the foundation for future in-depth characterization of aberrant value-based decision-making in mental disorders in the future.**

**p. 22:**

**Taken together, future use of innovative tools such as Influenca in large-scale, naturalistic assessments may provide a much more nuanced perspective on individual trajectories in decision-making and learning and their contribution to mental health.**

3. Additionally, the legend of Figure 3 is misleading, and it is not clear which colors represent A or B.

**We apologize for the unclarity of the legend. The color coding indicates which options the selected participant chose in each trial. The color orange indicates that they chose option A, whereas turquoise indicates that they chose option B. We now explicitly state that the observed option is color coded and changed the layout to better align the color with the corresponding option.**

4. Could you please provide the R or MATLAB code for the analysis?

**We are happy to provide MATLAB code for the model function used to fit the data as well as R Code used to calculate the ICCs and descriptive statistics. The code is shared in the following github repository: https://github.com/neuromadlab/ReliabilityInfluenca**

Reviewer #1:

**We thank the reviewer for their valuable suggestions that we gladly incorporated into the manuscript. We believe that those changes have improved the manuscript considerably.**

Abstract:
1. (minor) "Here, we developed..." -> "In this paper we present..."

**We have changed the wording in the abstract accordingly:**

2. (minor) "Notably, state items showed comparable ICCs as reinforcement learning parameters." -> It is not clear to me what the  term "state items" refers to.

**We thank the reviewer for pointing out that state items is not sufficiently specific. In the revised version, we use the term ‘physiological and mental states’ to refer to items assessing constructs such as hunger/satiety and mood before each run of the game.**

**p. 2:**

**In this paper, we present an open-source cross-platform application *Influenca* that provides a novel reward learning task complemented by ecological momentary assessment (EMA) of current mental and physiological states**

Method:
3. "Additional participants were recruited through social media, university mailing lists, and flyers. Moreover, participants in other studies of the lab are invited to play Influenca." -> How many participants were recruited from each population? Is it possible to evaluate what percentage of each population (i.e., mental disorders, healthy, etc.) played more than 10 runs and were therefore included in the analysis?

**We thank the reviewer for posing this important question. Since the game was freely available on our website and participants initially did not have to provide any information apart from consent to take part in the game, we do not have detailed information about the diagnoses of all participants. The majority of the recruited sample (314/392) completed a phone screening to determine physical and mental health for potential inclusion in lab-based studies, which included standardized diagnostic questions for mental disorders. Out of those 314 participants who completed the screening, 43 (14%) fulfilled the criteria for binge eating disorder. Moreover, after 10 runs, participants were invited to complete a set of questionnaires before they could unlock additional levels of the game. This included, for example, the Beck depression inventory (BDI, (Beck et al., 1996)) and a food addiction questionnaire that also provides a yes/no diagnosis for food addiction. Within this sample completing the questionnaires (357/392), 20% fulfilled the criteria for food addiction. BDI scores ranged from 0 to 49 with an average score of 11 (SD = 10.3). Using established BDI cut-offs, 42 participants (12%) had a mild depression, 33 (9.3%) a moderate depression, and 29 (8.2%) a severe depression. To better characterize the sample, we have now added more details to the sample description and a table with sample characteristics to the supporting information.**

**p. 8:**

**The sample was recruited across multiple studies and included participants with a wide range of BMI (14 – 58, MBMI = 26.4 kg²/m, SD ± 7.4) that was enriched for pathological eating and mood and anxiety disorder. Consequently, 43 participants fulfilled the criteria for a binge eating disorder and 107 participants reported symptoms of depression according to suggested cutoffs for the Beck Depression Inventory II (>= 14, Beck, Steer, Ball, & Ranieri, 1996, Table S1).**

4. The “virus”/”pandemic” theme might have lead to differences in participants’ behavior before and after the outbreak of the Covid-19 pandemic. Consider analyzing potential differences in participants behavior before 2020 and after 2020. If this is impossible, critically evaluate the potential influence in the limitations section.

**The reviewer raises an excellent point, and we agree that this unforeseen event might have changed behavior during the data collection. However, only a small fraction of the data was acquired before the onset of the pandemic. More precisely, only 8% (778 of 9729) of the runs were played before the first lockdown in Germany during the pandemic in March 2020 and only 18 (5%) participants played runs before and after onset of the pandemic.** **Notably, behavior across runs (i.e., increases in performance indicated by higher average rewards (t=5.9, p < .001), model fit (t=10.33, p < .001), reward sensitivity β (t=9.2, p <.001), and reliance on learned reward probabilities λ (t=3.03, p = .004), as well as lower learning rates for losses α_pun_ (t=-4.0, p = .0003). Nonetheless, we now include this information in the results section and discuss potential effects in the limitations section.**

**p. 8:**

**Of note, 42 participants (10%) started the game before March 2020, whereas the majority started playing after the onset of the COVID-19 pandemic.** **Still, participants starting before onset of the COVID-19 pandemic showed similar changes in behavior across runs (see S1 Appendix)**

**p. 26:**

**Last, to provide a gamified version of the task, we chose a framework conveying the basic mechanics of the task such as changing reward probabilities corresponding to changes in effectiveness of medication or additional levels associated with new pathogens that have to be treated. The design of the task was chosen considerably before onset of COVID-19 and even data collection started before the COVID-19 pandemic. Consequently, behavior might have changed considering the gamified content of fighting different viruses. Since the majority of participants started playing after the onset of the pandemic and reliability primarily evaluates potential changes in the rank order of participants, systematic effects of the pandemic on psychometric properties should be small in comparison other sources of variance.**

5. Reinforcement learning model: (p. 14) I understand why you would perform the following analyses based on the “best” model (i.e., the one which you identified beforehand). However, it might be worth considering to use one or a few alternative models in addition to the seemingly best one. I think that this would help to proof that the identified model really is the best one. One would not even have to report the analytical procedure of the alternative models, but rather state that all analyses have been run with different models (and model parameters), and the model with the two learning rates and the additive weighting performed best in all analyses.

**We thank the reviewer for this excellent suggestion. We have analyzed and reported the psychometric properties of the estimated parameters using the original model proposed by Behrens et al (2007) including only one learning rate and a multiplicative scaling of rewards and learning reward probability (1LR_Gamma). Crucially, the ICCs of the best fitting model are better:**

**Reward sensitivity:	ICC_beta_Behrens_ = .29	vs. ICC_beta_winning_model_ = .33
Log-likelihood:	ICC_loglik_Behrens_ = .32	vs. ICC_loglik_winning_model_ = .42
Win probability:	ICC_gamma_Behrens_ = .27	vs. ICC_lambda_winning_model_ = .40**

**Or comparable:**

**Learning rates:	ICC_alpha_Behrens_ = .55	vs. ICC_alphaPun_winning_model_ = .55**

**We have now added this analysis to the methods section and included a table with the results in the supporting information.**

**p. 12:**

**Crucially, parameters of the winning model showed better or similar reliability compared to the original model proposed by Behrens et al. (2007), suggesting also an improvement in psychometric criteria (Table A in S1 Appendix).**

6. Test-retest-reliability: This section seems rather long and elaborate, as test-retest-reliability is a common and well-known statistical test. I would recommend shortening the paragraph.

**We agree with the reviewer that most of the readers will be familiar with ICCs and other test-retest reliability measures and have streamlined this section. Nevertheless, there are different ICCs and formulas to calculate ICCs depending on the software, so we now provide details in the supporting information to support full transparency and replicability. Moreover, we would like to emphasize that psychometrics have only recently received more attention in the experimental literature because they are a limiting factor for the identification of potential behavioral markers of psychopathology** (Zorowitz & Niv, 2023)

**p. 15:**

**To assess the reliability of behavioral parameters and state items, we estimated ICCs using linear mixed-effects models (Raudenbush & Bryk, 2002). We report the ICC assessing absolute agreement as well as conditional ICCs considering systematic differences across runs (see supporting information). To evaluate which runs within the game provide the most reliable parameter estimates, we calculated the test-retest rank correlation (Spearman) for each (held out) run with the average of the parameter across all other runs. We interpreted the correlations according to recommendations by Taylor (1990) for correlation coefficients, where rs < .35 reflect low, rs between .36 and .67 reflect modest, rs > .67 reflect high correlations.**

Discussion:
7. “Since alterations [of value-based decision-making] have been linked to multiple mental disorders [...]” → The participants suffer, at least in part, from different mental disorders (as described in the method section). However, the inter-group differences between healthy participants and participants with binge eating disorders or depression have not been investigated. Would it not be interesting to see if the three “groups” differed regarding the quality of their value-based decision-making?

**We agree with the reviewer that differences in value-based decision-making between participants with different mental disorders are an important question of great interest. Consequently, in this manuscript, we first aim to establish that the gamified task in this smartphone-based, naturalistic setting has the psychometric qualities, such as a sufficient test-retest reliability across all runs, to investigate interindividual differences. One implicit assumption in this approach was that basic psychometric properties do not differ between groups, although groups might differ in how reliable their data is, partly due to symptoms of a disorder (e.g., impulse control). Therefore, we now calculated ICCs separately for groups of participants with a BDI<14 (i.e., likely no depression) vs. BDI=> 14 (i.e., likely presence of at least mild symptoms of depression). Likewise, we also explored differences in participants with binge eating disorder vs. no binge eating disorder. In line with the idea of largely invariant psychometric characteristics, ICC values were very similar across groups. Notably, the ‘psychopathology groups’ with a BDI=>14 as well as with binge eating disorder had higher ICCs for lambda (i.e., governing how reward and win probability are integrated; BDI<14: ICC = .37 vs. .43 in BDI=>14, no BED: ICC = .28 vs. .55 with BED). Of note, a higher ICC for lambda might also be explained by an increased variability between participants in participants with BED, as many participants across the whole sample show very high values for lambda (i.e., close to 1) for nearly all runs. In contrast, the ICC for the model fit was lower in the BED group (BED ICC = .35 vs. .42 in no BED). We have now added this information to the results section and included a table in the supporting information.**

**p. 18:**

**Notably, ICCs were comparable (Table S3) in both participants with vs. without depressive symptoms and participants with vs. without binge eating disorder, and ICCs for weighting of win probabilities, λ, were even higher in groups with psychopathology (BDI>=14: ICC = .43; BED: ICC = .55 vs. ICC = .37 in participants without those symptoms), indicating moderate reliability.** **However, this increase in ICC for λ might also be explained by an increased between participant variability in those smaller subsamples, as in the complete sample many participants show similarly high values throughout all runs.**

8. (minor) “to boost psychometric properties” → While I like the enthusiastic connotation, I would recommend choosing another term.

**We have now replaced “boost” with “improve”:**

**p.22:**

**highlighting the benefit of multi-run assessments to improve psychometric properties.**

9. (minor, general) "Run-based data that support the findings of this study will be publicly shared on osf upon publication." While this might be a standard phrase to inform reviewers/readers about data availability, it sounds as if the authors only share data that supports the findings of the study while keeping conflicting findings to themselves. I am sure that this is not the case, but it might be a good idea to reword this sentence.

**We apologize for using a potentially misleading statement and have rephrased the statement accordingly:**

**p. 28:**

**Run-based data, including maximum-likelihood estimates of the reported results, will be publicly shared on osf upon publication.**

Reviewer #2: The manuscript by Neuser et al. provides a valuable insight into valuable-based decision-making and its measurement methods. Nevertheless, I would like to suggest some ideas and questions for further improvement. All suggestions are grouped according to the respective section.

**We thank the reviewer for their positive evaluation of our manuscript and their thoughtful comments. We are confident that our revisions improve conciseness, making it more accessible for a broad readership.**

Introduction:
1. The plethora of statistical parameters of the referenced studies could be omitted for the sake of conciseness

**We thank the reviewer for this valuable suggestion and have considerably shortened the section about different ICCs values. In the revised version, we focus more on the important take-away messages now:**

**p. 5:**

**To interpret individual trajectories, including markers of clinical progression or potential effects of interventions (Dubois & Adolphs, 2016), a sufficient test-retest reliability is necessary. Consequently, the lack of formal assessments of reliability might hamper the widespread application of reinforcement learning tasks to study associations with psychopathology (Rodebaugh et al., 2016) (Zorowitz & Niv, 2023). Reliability levels for decision-making tasks typically vary from low to moderate (Buelow & Barnhart, 2018). In reinforcement learning tasks, parameter estimates of computational models also exhibit low to moderate reliability (Moutoussis et al., 2018; Santesso et al., 2008; Schaaf, Weidinger, Molleman, & van den Bos, 2023). Tasks related to probabilistic reinforcement learning have shown comparably low reliabilities in clinical samples as well (Pratt et al., 2020; Shiner et al., 2012). Preliminary evidence has shown that reliabilities do not differ between online and lab-based assessments (Weidinger, Gradassi, Molleman, & van den Bos, 2019) or between raw dependent variables and latent variables (Enkavi et al., 2019).** **Furthermore, test-retest reliability does not only depend on the task itself, but may be improved by careful experimental planning (Mkrtchian, Valton, & Roiser, 2021; Zorowitz & Niv, 2023) or hierarchical modeling approaches, such as hierarchical Bayesian estimations (Brown, Chen, Gillan, & Price, 2020), integrating repeated measures into the generative model (Haines et al., 2020; Waltmann, Schlagenhauf, & Deserno, 2022). Likewise, integrating response times in addition to choices may improve reliability (Shahar et al., 2019), although such approaches are not commonly used in clinical samples yet. Taken together, despite the widespread use of reward learning tasks, the reliability of individual differences related to value-based decision-making and reward learning is still underreported, especially across many runs in naturalistic settings (Zech et al., 2022) that would enable monitoring of individual trajectories at scale.**

2. The content in this section could be more balanced in terms of reliability and psychological elements (e.g. psychobiological process, biomarkers). I would suggest to reduce the parts for reliability to eventually strengthen the conciseness

**We agree with the reviewer that the emphasis on reliability was likely suitable for a narrower audience than the potential readership of PLoS Digital Health. The app was specifically designed to assess individual fluctuations in reinforcement learning over time in combination with transdiagnostic symptoms such as pathological eating behavior or anhedonia. To provide a basis for in-depth characterization of reward learning along such dimensions of psychopathology, the manuscript provides important psychometric information on behavior across a typical sample with mixed symptoms. We envision that our work will kindle future efforts towards partitioning differences in value-based behavior into trait- and state-like characteristics, as we have done in other work using high-dimensional data** (e.g., Kühnel et al., 2022)**. In addition to streamlining the paragraph about reliability, we have now further contextualized the manuscript regarding psychological elements and mental disorders.**

**p. 6:**

**To summarize, cross-sectional measures provide a snapshot of value-based decision-making, which cannot separate trait-like differences from state-like differences in behavior, specifically if reliability is low. To provide a much more comprehensive estimation of reliability in a clinically relevant setting, we present densely sampled longitudinal data of reinforcement learning and decision-making in a sample with diverse symptoms of psychopathology (open-source app Influenca: [www.neuromadlab.com/en/influenca-2](http://www.neuromadlab.com/en/influenca-2)). To facilitate the investigation of altered value-based decision making in participants with pathological eating behavior (e.g., restrictive eating or binge eating) or mood and anxiety disorders, we developed a smartphone-based, gamified assessment. We measured decision-making over extended time periods in a large sample recruited to span a large range of body mass index (BMI) with diverse symptoms of psychopathology. The longitudinal assessment across 30 runs enables us to investigate how reinforcement learning changes over runs and how variable behavior is within participants. Such a dense sampling approach provides important new information beyond a lab-based snapshot of behavior to characterize individual trajectories of behavior. For example, binge eating might be characterized by increased fluctuations in reward sensitivity that are associated with higher fluctuations in reward learning (Neuser, Kühnel, Svaldi, & Kroemer, 2020). Here, we first evaluate the psychometric properties, such as reliability of behavioral parameters, to ensure that the data provided by the app is able to provide reliable trait-like inter-individual differences while still capturing meaningful intra-individual variability. Such intra-individual variability may reflect potential adaptations in individual behavior, for example, in response to treatments, reflecting the range of the individual repertoire of behavior. Hence, more detailed knowledge of the psychometric characteristics of these parameters will provide the foundation for future in-depth characterization of aberrant value-based decision-making in mental disorders.**

Methods:
3. It is not clear why participants with binge eating disorder and depression, as well as healthy participants could join the study. This should be explained, maybe with the help of mentioning further exclusion criteria. Furthermore, it should be stated why different groups of participants could join the study and why these subgroups were not evaluated respectively.

**The reviewer raises an important point that we now elaborate on. An advantage of an online assessment of behavior is that it provides better access to populations that may not participate in repeated lab visits as part of a study. Hence, such an approach offers the opportunity to quickly gather large and clinically diverse datasets that are necessary for the evaluation of robust behavioral markers of mental disorders or transdiagnostic symptoms (Gillan et al., 2016; Hauser et al., 2022; Paulus & Thompson, 2021). By design, we did not enforce exclusion criteria for the online assessment, but relied on deep phenotyping via questionnaires and a phone interview to increase the generalizability of findings to settings outside of the lab. For example, most mental disorders are comorbid with other conditions classical case–control studies need to use exclusion criteria instead of modeling comorbidities as they occur in clinical populations (Gillan & Rutledge, 2021). Therefore, we gathered data from participants and enriched it for psychopathology related to eating disorders and mood and anxiety disorder to evaluate the psychometric quality of the assessment in the intended target population. Many studies reporting psychometric indices of reinforcement learning tasks so far have suffered from comparatively small sample sizes, hindering replicability of the results (Hauser et al., 2022) due to the uncertainty of obtained estimates (see e.g., Fröhner et al., 2019 for a resampling approach that demonstrates that larger samples are necessary to obtain precise point estimates for the reliability of biomarkers). Therefore, we included all participants who passed a quality control of their behavioral data in this analysis to report representative estimates of psychometric properties of the task in a clinically diverse population. In the revised version, we now emphasize our objectives and sample recruitment in the methods section. Moreover, we now report reliability data also separately for participants with vs. without symptoms of depression as well as with vs. without binge eating disorder.**

**p. 8:**

**Second, participants were provided with a second informed consent form before completing online questionnaires after completing Run 10.** **The app was included in two studies in healthy participants and patients, focusing on binge eating disorder with and without comorbid mood and anxiety disorders (n = 316) and major depressive disorder (n=65). Participants received a fixed compensation (€20) if they completed the online assessment. In the study on binge eating disorder, the online assessment including the app was part of a module to acquire data from participants with pathological eating behavior and binge eating episodes (subjective or objective). To ensure a wide range of symptoms of psychopathology, we did not exclude participants based on the presence of a mental disorder. Additional participants were recruited via the same channels such as social media, university mailing lists, and flyers without emphasizing the emphasis on symptoms of eating disorders or mood and anxiety disorders. Likewise, we invited participants contacting our lab to complete this online assessment including Influenca.**

**p. 18:**

**Notably, ICCs were comparable (Table S3) in both participants with vs. without depressive symptoms and participants with vs. without binge eating disorder and the ICC for weighting of win probabilities, λ, were even higher in groups with psychopathology (BDI>= 14: ICC = .43; BED: ICC = .55 vs. ICC .37 in participants without those symptoms)**

4. Why had participants to play at least 10 runs to be included?

**We thank the reviewer for this question and are sorry that we did not explain this element of the design before. In this analysis, we only included participants with at least 10 completed runs so that we were able to estimate individual reliability across runs and determine potential changes over repeated measurements. We selected Level 10 because we only collected online questionnaires including demographic information on the participants if they wanted to continue playing the game. Therefore, a high proportion of participants completed at least 10 levels, whereas some participants stopped earlier, particularly after doing just one Run (Figure S1). We have added the rationale for this decision to the methods section.**

**p.8:**

**To estimate changes in reinforcement learning parameters and their test-retest reliability across repeated runs, we included participants who completed at least 10 runs of Influenca after passing quality control (i.e., 106 runs were excluded due to random choices: log-likelihood < -100.13, Figure S2) in the current analysis.**

5. Why was the storyline / narrative of Influenca, a clinical topic, chosen? Especially since participants with psychiatric disorders were included in the study. Is there a coherence between these domains?

**To provide a compelling, gamified framework for the task, we needed to develop a narrative that helps convey the basic mechanics of the game without imposing incorrect assumptions about the contingencies. After discussing several potential storylines, we chose the topic of selecting the best medication because it would convey important elements in a relatable manner to a wide audience. For example, most people understand that pathogens mutate over time and that this is not fully predictable, which may change the effectiveness of a given medication. In addition, we felt that this storyline would facilitate the independence of runs while communicating a clear reward schedule (depending on the success of the medication) with the opportunity to progress over runs. Illustratively, we already designed a ‘call for action’ as part of the storyline to save people by delivering the right medication. When we designed the task, we could not anticipate the onset of the COVID-19 pandemic at such a scale and we then adjusted the advertisement slightly to avoid having a larger conceptual overlap with the specifics of the COVID-19 pandemic.**

6. Are there two versions of the app? The description of the app gives the impression that there is one gamified version and one version without game design elements.

**We apologize for the potentially misleading phrasing. Indeed, there is only a gamified version of the app, which presents a gamified narrative for the reinforcement learning task. We used the tag of a gamified version in reference to typical lab-based tasks that often do not provide a coherent narrative. To improve clarity, we have rephrased the sentence in the revised version.**

**p. 10:**

**In the Influenca app, a gamified version of a classic reinforcement learning task, participants had to fight a virus pandemic by finding the most effective medication and successfully treating as many people as possible.**

7. I would suggest introducing more precisely, why it was important to create different reinforcement learning models. Is it relevant for the statement of the paper to mention the models in such detail? Does it fit with the remaining sections?

**In hindsight, we agree with the reviewer that this section includes a lot of information that is not necessary for understanding the paper and more of interest for a narrower readership. Still, performing rigorous model comparisons is an integral step of computational modeling, even if a task is similar to previously published work** (Behrens et al., 2007)**. First, based on simulations of parameter recovery and performance measures, our task included the loss of points after incorrect decisions instead reward omission. Second, behavior in our gamified online version might differ from the performance in the original publication. Third, previous publications based on comparable tasks found different models to best describe the data** (Behrens et al., 2007; Gagne et al., 2020)**. Therefore, it is recommended that each study performs and reports model comparisons (Wilson & Collins, 2019). Nevertheless, we agree that the section should be more accessible to a broad readership, so we now describe the final model (including formulas for replicability) and moved additional information about model comparisons to the supporting information. Moreover, we explain how each parameter changes behavior of an agent so that a broader readership can evaluate the conceptual meaning of each parameter. In our opinion, this is necessary to assess differences or changes in reinforcement learning and relate them to symptoms across various disorders.**

**p. 12:**

***Reinforcement learning model***

**To model different facets of reward learning, we used choice data from individual runs and fit a reinforcement learning model with two learning rates (α), reward sensitivity (β), and a parameter determining the additive weighting of reward magnitude and win probability during the choice. To ensure that our model was suitable for the data** (Wilson & Collins, 2019)**, the model was chosen after model comparison (for details, see SI) between previously described models for this task (Behrens et al., 2007; Gagne, Zika, Dayan, & Bishop, 2020; Neuser, Kühnel, Svaldi, & Kroemer, 2020). Crucially, all estimated parameters of the selected model showed better or similar reliability compared to the original model proposed by Behrens et al. (2007), suggesting also an improvement in psychometric criteria (Table S2). In the implemented reinforcement learning model, participants are assumed to decide between the options in each trial based on the inferred win probability of each option. These probability estimates, *p_win_*, are learned, by updating the estimated win probability after each trial following a simple delta rule:**

$\boldsymbol{p}_{\boldsymbol{win,t+1}} \left( \boldsymbol{Option A} \right)\boldsymbol{=}\boldsymbol{p}_{\boldsymbol{win,t}} \left( \boldsymbol{Option A} \right)\boldsymbol{+ \alpha*RP}\boldsymbol{E}_{\boldsymbol{t}}\boldsymbol{(Option A)}$	**(1)**

**where α ∈ [0, 1] denotes the learning rate and the RPE the reward prediction error comparing the expected win probability with choice outcome, that is scaled by the learning rate.**

$\boldsymbol{RP}\boldsymbol{E}_{\boldsymbol{t}}\left( \boldsymbol{Option A} \right)\boldsymbol{=}\boldsymbol{r}_{\boldsymbol{t}}\boldsymbol{-}\boldsymbol{p}_{\boldsymbol{win,t}}\boldsymbol{(Option A)}$	**(2)**

$\boldsymbol{r=}\left\{ \begin{aligned} \boldsymbol{1 if Option A results in win} \\ \boldsymbol{0 if Option A results in loss} \end{aligned} \right.$	**(3)**

**The learning rate therefore quantifies how quickly an individual updates choice preference with changing outcome contingencies. In other words, high learning rates lead to quick updates by putting more weight on recent choice outcomes. In contrast, low learning rates lead to slow updates by putting less weight on recent choice outcomes and more weight on former feedback, leading to smoother changes in estimated reward probabilities (Figure 2b).**

**In our winning model, we also included separate learning rates for wins and losses (Aylward et al., 2019; Gagne et al., 2020). Choices in each trial are then generated by combining the estimated win probability of the options (**$\boldsymbol{p}_{\boldsymbol{win}} \left( \boldsymbol{Option A} \right)$**) with the associated reward values of each option (*f*) (**$\boldsymbol{f}_{\boldsymbol{OptA}} \boldsymbol{\in}\left[ \mathbf{0, 100} \right]\mathbf{and}\boldsymbol{f}_{\boldsymbol{B}} \boldsymbol{\in}\left[ \mathbf{0, 100} \right]\boldsymbol{with}\boldsymbol{f}_{\boldsymbol{A}}\boldsymbol{=100-}\boldsymbol{f}_{\boldsymbol{B}}$**).** **In our winning model, both estimated win probability and reward magnitude are additively weighted (Gagne et al., 2020). This means that both the difference in win probability between the options and the difference in points between options are first scaled by a weighting parameter, λ, and then summed:**

$\boldsymbol{W}_{\boldsymbol{t}}\left( \boldsymbol{Opt. A} \right)\boldsymbol{= \lambda}\left( \boldsymbol{p}_{\boldsymbol{win,t}}\left( \boldsymbol{Opt.A} \right)\boldsymbol{-}\left( \boldsymbol{1-}\boldsymbol{p}_{\boldsymbol{win,t}}\left( \boldsymbol{Opt.A} \right) \right) \right)\boldsymbol{+}\left( \boldsymbol{1- \lambda} \right)\left( \boldsymbol{f}\left( \boldsymbol{Opt.A} \right)\boldsymbol{-f}\left( \boldsymbol{Opt. B} \right) \right)$ **(4)**

**Here, λ is constrained between 0 and 1 and low values of the weighting parameter (Figure 2c upper panel) lead to decisions based on reward magnitude whereas high values (Figure 2c lower panel) lead to decisions based on win probability. Last, the probability of choosing option A is computed with a sigmoid probability function based on the weighted sum of win probability and reward points.**

$\boldsymbol{p}_{\boldsymbol{choice,t}} \left( \boldsymbol{Option A} \right)\boldsymbol{=}\frac{\mathbf{exp}\boldsymbol{(}\boldsymbol{W}_{\boldsymbol{t}} \left( \boldsymbol{Option A} \right)\boldsymbol{* \beta)}}{\boldsymbol{(}\exp\left( \boldsymbol{W}_{\boldsymbol{t}} \left( \boldsymbol{Option A} \right)\boldsymbol{* \beta} \right)\boldsymbol{+}\mathbf{exp}\boldsymbol{(}\boldsymbol{W}_{\boldsymbol{t}} \left( \boldsymbol{Option B} \right)\boldsymbol{*\beta))}}$	**(5)**

**Here, the reward sensitivity, *β* ∈ [0, Inf], scales the estimated weight of an option (i.e., weighted sum of win probability and points) with an individual factor indicating the subjective value. The reward sensitivity determines the predictability of value-based decisions in such a way that high reward sensitivity (Figure 2a, light blue colors have more extreme choice probabilities) leads to very predictable choices based on the weights, while low reward sensitivity (Figure 2a, dark blue colors have less clear choice probabilities) leads to noisier decisions (Neuser et al., 2020). This parameter is also referred to as inverse temperature and simultaneously captures choice stochasticity (Huys et al., 2013).**

8. Do the learning models include the states captured via EMA?

**Since we collected state ratings and compared the ICCs to model parameters, this is an excellent question. In this manuscript, we estimate the parameters of each run independently using maximum likelihood estimation to derive values that correspond conceptually to cross-sectional studies with few runs. These estimates do not depend on or include any state measurements in the estimation procedure. In this manuscript, we only investigate changes and fluctuations across runs, not how subjectively rated states may influence reinforcement learning parameters. Notwithstanding, potential associations of learning parameters with momentary states is an important question for future research, which is better addressed by hierarchical Bayesian models, however. Since such models do not provide single-run estimates that can be easily compared to conventional benchmarks for psychometric quality of an instrument, we decided to separate the questions to provide optimized answers for each question.**

**p. 26:**

**Fourth, apart from the rich possibilities provided by extensions of the computational models, future work should focus on the reciprocal influence of momentary states (including metabolic states such as hunger) and parameters of value-based decision-making (Blain & Rutledge, 2020; Rutledge et al., 2017) to differentiate meaningful fluctuations in reward learning behavior from measurement noise that leads to reduced reliability. To this end, hierarchical Bayesian models can be extended to directly incorporate the metabolic or mood state of each run to then modulate RL parameters (Swart et al., 2017).**

Results:
9. Both sections, results and methods, include sophisticated content and might not be easy to understand for the broader audience. As the journal addresses the broader audience, I would suggest simplifying these sections.

**We thank the reviewer for this thoughtful suggestion and agree that some of the details regarding the model building are not necessary for a broader readership. Therefore, we moved technical details of the model description and model comparison to the supplementary information to provide this information for expert readers while streamlining the paper for a more diverse audience, including more clinically interested readers. Nevertheless, we think that all components of the results are helpful to understand and evaluate the results and their scope regarding the assessment of inter-individual differences in a clinically relevant domain that has received more limited attention for longitudinal assessments so far. Still, we have revised the results section for better emphasis to improve clarity (e.g., by moving details on specific estimates to the supporting information). Illustratively, we now state the purpose of describing optimal behavior within the task and better relate the numerical results to behavioral differences on the task. This is an important aspect as even small changes in the setup of the task compared to previous versions may change which behavior is most beneficial. Such a comparison to optimal behavior is also important to interpret inter-individual differences with regard to psychopathology.**

**p. 16:**

**In each run, participants made 150 choices between two options of varying reward value and fluctuating win probabilities that had to be inferred over time. To estimate reinforcement learning parameters from value-based choices, we applied computational modeling. First, we analyzed which behaviors (i.e., parameter combinations) were associated with high average reward rates to determine optimal behavior. An increased average reward was reached with moderate learning rates for losses, α_loss_ between 0.2 – 0.275 (all *p*s against other ranges, binned with .075 width < .05, Figure 2b). This indicates that not shifting from the preferred option in response to single negative outcomes is advantageous to track “true” fluctuations in the hidden random walks, as a high learning rate (Figure 2b, top panel) leads to fast switches in choices due to recent events. As found to be optimal in the task, a lower learning rate (Figure 2b, bottom panel) reflect more patience in light of surprising outcomes, reducing the number of switches between preferred options. In contrast, the learning rate for wins barely influenced the average reward (Figure 2c) as the task was designed to accommodate a large range of behavior without penalizing a given strategy. Moreover, our data shows a clear association of higher average rewards with higher reward sensitivities, although the improvement plateaus around the upper limit of our simulation (β > 5). Since reward sensitivity reflects the predictability of choices based on inferred differences in values, a high value indicates that successful participants make more deterministic choices even if there are only small relative differences in inferred value between the options. Last, making decision purely based on the difference in win probability while disregarding differences in potential reward as indicated by higher λ values was associated with the highest average rewards (Figure 3e) and almost all participants showed a very high λ.**

10. Thought could be given to waiving the detailed description of creating the models in favor of embedding the results into the broader context of psychiatric disorders with a reference to the study population. Especially as the discussion starts with a reference to psychiatric disorders.

**We thank the reviewer for this suggestion and agree that the results section still contained some non-essential details regarding the analyses or secondary results of control analyses. To increase appeal to a broader audience, we have now streamlined the manuscript using a stronger focus on key results and their implication. Regarding the contextualization of our work, the main goal of the study is to validate the behavioral measures provided by the app using common psychometric indices. Such a verification of the basic mechanics of the task is a prerequisite for investigating interindividual differences, especially in longitudinal settings. Moreover, we recruited a clinically diverse sample to ensure that psychometric properties are suitably high in the target population for repeated assessments of value-based decision-making and we now additionally report reliability estimates for subgroups of the sample.**

**p. 6:**

**To summarize, cross-sectional measures provide a snapshot of value-based decision-making, which cannot separate trait-like differences from state-like differences in behavior, specifically if reliability is low. To provide a much more comprehensive estimation of reliability in a clinically relevant setting, we present densely sampled longitudinal data of reinforcement learning and decision-making in a sample with diverse symptoms of psychopathology (open-source app Influenca: www.neuromadlab.com/en/influenca-2). To facilitate the investigation of alterated value-based decision making in participants with pathological eating behavior (e.g., restrictive eating or binge eating) or mood and anxiety disorders, we developed a smartphone-based, gamified assessment. We measured decision-making over extended time periods in a large sample recruited to span a large range of body mass index (BMI) and with diverse symptoms of psychopathology. The longitudinal assessment across 30 runs enables us to investigate how reinforcement learning changes over runs and how variable behavior is within participants. Such a dense sampling approach provides important new information beyond a lab-based snapshot of behavior to characterize individual trajectories of behavior. For example, binge eating might be characterized by increased fluctuations in reward sensitivity are associated with higher fluctuations in reward learning (Neuser, Kühnel, Svaldi, & Kroemer, 2020). Here, we first evaluate the psychometric properties, such as reliability of behavioral parameters to ensure that the data provided by the app is able to provide reliable trait-like inter-individual differences while still capturing meaningful intra-individual variability. Such intra-individual variability may reflect potential adaptations in individual behavior, for example, in response to treatments, reflecting the range of the individual repertoire of behavior. Hence, more detailed knowledge of the psychometric characteristics of these parameters will provide the foundation for future in-depth characterization of aberrant value-based decision-making in mental disorders in the future.**

**p. 8:**

**The app was included in two studies in healthy participants and patients, focusing on binge eating disorder (n = 316) and major depressive depression (n=65). Participants received a fixed compensation (€20) if they completed the online assessment. In the study on binge eating disorder, the online assessment including the app was part of a module to acquire data from participants with pathological eating behavior and binge eating episodes (subjective or objective). To ensure a wide range of symptoms of psychopathology, we did not exclude participants based on the presence of a mental disorder. Additional participants were recruited via the same channels such as social media, university mailing lists, and flyers without emphasizing the emphasis on symptoms of eating disorders or mood and anxiety disorders. Likewise, we invited participants contacting our lab to complete this online assessment including Influenca.**

**p. 18:**

**ICCs were comparable (Table S3) when analyzing participants with vs. without depressive symptoms or with vs, without binge eating disorder separately, although the ICC for weighting of win probabilities, λ, were higher in groups reporting depressive symptoms (ICC = .43) or binge eating disorder (ICC = .55).**

Discussion:
11. It should be discussed why the storyline of Influenca is used for participants with psychiatric disorders (as already mentioned in the methods section)

**We thank the reviewers for their valuable suggestion. Briefly, we decided for this scenario as it provides a narrative that integrates the main mechanics of the task: 1) Changes in win probability corresponding to changes in effectiveness of the medication. 2) New levels / runs that are independent from each other corresponding to new viruses. 3) Variability in points won corresponding to the number of people saved. When we designed the task, we could not anticipate the onset of the COVID-19 pandemic at such a scale and we then adjusted the advertisement slightly to avoid having a larger conceptual overlap with the specifics of the COVID-19 pandemic. We now added our rational and limitations corresponding to the onset of the COVID-19 pandemic to the discussion.**

**p. 26:**

**Last, to provide a gamified version of the task, we chose a framework conveying the basic mechanics of the task such as changing reward probabilities corresponding to changes in effectiveness of medication or additional levels associated with new pathogens that have to be treated. The design of the task was chosen considerably before onset of COVID-19 and even data collection started before the COVID-19 pandemic. Consequently, behavior might have changed considering the gamified content of fighting different viruses. Since the majority of participants started playing after the onset of the pandemic and reliability primarily evaluates potential changes in the rank order of participants, systematic effects of the pandemic on psychometric properties should be small in comparison other sources of variance.**

12. A latency of at least 2 hours was stated to secure for different metabolic states, but can this be guaranteed? Was it somehow controlled? Otherwise, this should be discussed.

**The reviewer raises an excellent point. Indeed, the software checked whether the 2h had passed, but we did not impose any restrictions on how similar the ratings of the state should be. The idea of this setting was to discourage repeated runs in more or less the same state, and we reasoned that imposing such a constraint would facilitate sampling of various states throughout the study, even if they might be qualitatively similar. In other words, this was implemented to reduce systematic differences in how similar behavior would be due to differences in latency. To give the reviewers and editor a better idea of the distribution over runs, we analyzed the individual ranges in hunger and satiety ratings across all levels. Most participants (i.e., >90%) reported hunger ratings spanning a range of at least 50 points and satiety ratings spanning range of at least 60 points (Figure R.1), indicating that levels were indeed played across various metabolic states at (somewhat) independent time points. We have also added this figure to the supporting information.**

**Figure R.1:** Participants completed Influenca levels across a variety of metabolic states. a)-b) distribution of individual ranges (i.e., maximal value – minimal value) of hunger (a) and satiety (b) ratings show that more than 90% participants had ranges exceeding 50 or 60 points for hunger and satiety, respectively. c)-d) Density of run-based hunger and satiety ratings for each participant show that large areas of possible metabolic states are covered in most participants.

**p. 26:**

**Likewise, multi-run assessments conducted across various mental or metabolic states (see S6 Figure for distributions) part may provide a better approximation of generalizable inter-individual differences in value-based decision-making compared to the typical single-run assessments in the lab after limited practice on the task.**
